# The Resistin/TLR4/miR-155-5p axis: a novel signaling pathway in the onset of hypothalamic neuroinflammation

**DOI:** 10.1186/s12974-025-03522-3

**Published:** 2025-08-04

**Authors:** Marianne Prévost, Delphine Crépin, Sarah Al Rifai, Ghislaine Poizat, Mélanie Gonçalves, Femke van Barneveld, Rozhina Shadpay, Karim Taouis, Laure Riffault, Yacir Benomar, Mohammed Taouis

**Affiliations:** 1 Paris-Saclay Institute of Neurosciences (NeuroPSI), UMR 9197, University of Paris-Saclay, CNRS, 151 route de la Rotonde, Saclay, F-91400 France; 2 University of Paris-Saclay, CNRS/IN2P3, IJCLab, Orsay, 91405 France; 3 Laboratoire de Génétique et Biologie Cellulaire, University of Versailles St-Quentin-en- Yvelines, Montigny-Le-Bretonneux, Paris-Saclay, France; 4 University of Wageningen, Wageningen, Netherlands

**Keywords:** Hypothalamic neuroinflammation, Resistin, miR-155-5p, High fat diet, Glucose intolerance

## Abstract

Hypothalamic neuroinflammation plays a pivotal role in the development of metabolic disorders, contributing to obesity and insulin resistance. Hypercaloric diets rich, particularly high-fat diets (HFDs) induce hypothalamic neuroinflammation, which has been shown to precede peripheral inflammation, even after short-term exposure. However, the mechanisms initiating this central inflammatory response, especially the mediators involved, remain incompletely understood. In this study, we demonstrate that HFD consumption induces the expression of resistin in the hypothalamus. Resistin, an adipokine known to promote inflammation and insulin resistance, was found to modulate the expression profile of microRNAs in the hypothalamus. Notably, intracerebroventricular administration of resistin led to the upregulation of miR-155-5p in a TLR4-dependent manner. Consistently, HFD feeding elevated miR-155-5p levels in the mediobasal hypothalamus. Given the established role of miR-155-5p in promoting macrophage activation in peripheral tissues, we examined its expression in microglial cells. We found that both resistin and palmitate, a saturated fatty acid that mimics HFD-induced metabolic stress, increased miR-155-5p expression in the SIM-A9 microglial cell line, which is derived from mouse cerebral cortex tissue without transformation or artificial immortalization. Our findings reveal a novel resistin/TLR4/miR-155-5p signaling axis that may initiate hypothalamic neuroinflammation. Moreover, we show that the induction of miR-155-5p by resistin depends on activation of the NF-κB, JNK, and p38 MAPK signaling pathways. To further explore this pathway, we performed High-Throughput Sequencing of RNA isolated by Cross-Linking Immunoprecipitation (HITS-CLIP) to identify miR-155-5p targets in SIM-A9 microglia and in the hypothalami of both male and female mice. This approach revealed several common targets, including *Quaking* and *Elmo1*, genes implicated in microglial phagocytosis and engulfment. These targets were validated, as transfection of SIM-A9 cells with a miR-155-5p mimic led to downregulation of their expression. Importantly, hypothalamic knockdown of miR-155-5p in both male and female mice improved glucose tolerance and restored *Quaking* expression in hypothalamic microglia. In conclusion, our data show that HFD promotes hypothalamic resistin expression, which in turn upregulates miR-155-5p via TLR4 and downstream signaling pathways, thereby contributing to hypothalamic neuroinflammation and disrupted glucose homeostasis. We identify the resistin/TLR4/miR-155-5p axis as a novel and critical pathway in the early events of diet-induced neuroinflammation.

**Supplementary Information:**

The online version contains supplementary material available at 10.1186/s12974-025-03522-3.

## Background

The mechanisms underlying the onset of hypothalamic inflammation remain poorly understood. Here, we identify a novel signaling pathway involved in the early stages of HFD-induced hypothalamic neuroinflammation, comprising resistin, TLR4, and miR-155-5p. Specifically, HFD increases the expression of hypothalamic resistin, which in turn activates the TLR4 pathway, leading to the upregulation of miR-155-5p. This is mediated through the NF-κB, JNK, and p38 signaling pathways. Moreover, we identify miR-155-5p targets in a microglial cell line and in the hypothalamus, notably including Quaking, a protein that plays a key role in microglial function. Our findings open new avenues for understanding HFD-induced hypothalamic inflammation by highlighting the role of hypothalamic resistin and uncovering miR-155-5p targets that may serve as potential therapeutic candidates.

## Introduction

Neuroinflammation in the hypothalamus has been extensively implicated in the pathogenesis of various metabolic disorders, including obesity, insulin resistance, and type 2 diabetes [1, 2]. Emerging evidence suggests that, similar to peripheral inflammation, hypothalamic neuroinflammation plays a crucial role in energy homeostasis and metabolic regulation disruption. This neuroinflammatory state is exacerbated by chronic consumption of hypercaloric diets, particularly high-fat diets (HFD), which induce metabolic stress and activate resident immune cells, such as hypothalamic microglia, in a process known as microgliosis. This activation leads to a sustained upregulation of pro-inflammatory cytokines, thereby perpetuating a cycle of inflammation and metabolic dysfunction [3–6]. Additionally, prolonged exposure to an HFD has been associated with macrophage infiltration into the hypothalamic region, further amplifying the local immune response and exacerbating neuroinflammatory conditions [7].

Beyond its central effects, HFD consumption significantly impacts peripheral metabolic tissues, particularly adipose tissue, where it induces a state of chronic inflammation and disrupts the secretion of adipokines. Among these adipokines, leptin and resistin have attracted considerable attention due to their roles in modulating energy balance, glucose metabolism, and inflammatory responses [8]. Leptin, a well-characterized hormone, is primarily known for its regulatory role in appetite and energy expenditure, yet it also contributes to immune signaling and inflammatory pathways. Conversely, the physiological role of resistin remains less well understood, despite mounting evidence implicating it in inflammatory and metabolic processes. In rodents, resistin is predominantly secreted by adipose tissue, whereas in humans, it is primarily produced by immune cells, suggesting a potential species-specific functional divergence [9].

Notably, elevated plasma resistin levels have been strongly correlated with systemic inflammation and insulin resistance in rodents and humans, reinforcing the hypothesis that resistin may be a molecular link between obesity and metabolic dysfunction [10]. Intriguingly, resistin mRNA expression has been detected in the hypothalamus [11], although its exact role within this brain region remains poorly defined. Prior studies from our research group demonstrated that intracerebroventricular (ICV) administration of resistin induces significant hypothalamic neuroinflammation, leading to impaired neuronal insulin signaling and systemic insulin resistance in key metabolic tissues such as the liver, muscle, and adipose tissue. Our investigations further identified Toll-like receptor 4 (TLR4) as a critical receptor mediating resistin’s effects, revealing that resistin-induced hypothalamic neuroinflammation operates through TLR4-dependent mechanisms [12, 13].

At the molecular level, resistin is known to activate the nuclear factor kappa B (NF-κB) signaling pathway, a key regulator of inflammatory gene expression, thereby enhancing the production of pro-inflammatory cytokines and promoting glial cell activation. These processes collectively contribute to hypothalamic inflammation, insulin resistance, and metabolic dysregulation [14, 15]. Given these findings, it is becoming increasingly clear that resistin exerts its pro-inflammatory actions in the hypothalamus via NF-κB and TLR4 signaling pathways, ultimately leading to metabolic disorders. However, the precise molecular mechanisms governing resistin’s role in the hypothalamus remain largely unexplored. While resistin expression in adipose tissue is closely associated with body fat mass, its regulation in the hypothalamus in response to obesogenic conditions, such as chronic HFD consumption, remains an open question.

Given the profound impact of central resistin on neuroinflammatory pathways, we hypothesize that resistin modulates the hypothalamic transcriptome by altering microRNA (miR) expression. MiRs are small, non-coding RNAs that function as potent post-transcriptional regulators of gene expression. Studies suggest that miRs regulate approximately 60% of protein-coding genes in the human genome [16, 17], influencing gene expression through mechanisms such as mRNA degradation or translational inhibition without directly modifying mRNA levels. Notably, dysregulated miR expression has been linked to numerous pathological conditions, including metabolic disorders, inflammatory diseases, and cancer, highlighting their significance as key molecular mediators [18–20]. Previous research has established miR-155-5p as a key regulator of macrophage activation, immune cell function, and inflammation in peripheral tissues [21–23]. However, its function in hypothalamic microglia remains largely unknown, prompting us to investigate its regulatory role in this context.

In this study, we demonstrate that high-fat diet (HFD) consumption increases resistin expression in the hypothalamus and that resistin upregulates miR-155-5p expression in a Toll-like receptor 4 (TLR4)-dependent manner. We further identified miR-155-5p target genes in a microglial cell line and the hypothalamus, revealing genes primarily involved in microglial function. Finally, knockdown of miR-155-5p in the hypothalamus improved glucose tolerance in HFD-fed mice. Our findings identify the resistin/TLR4/miR-155-5p axis as a novel mechanism contributing to hypothalamic inflammation and microglial activation.

## Results

### HFD induces inflammation, microglia activation, overexpression of resistin in the hypothalamus, and impairs whole-body insulin sensitivity

**Fig. 1:**
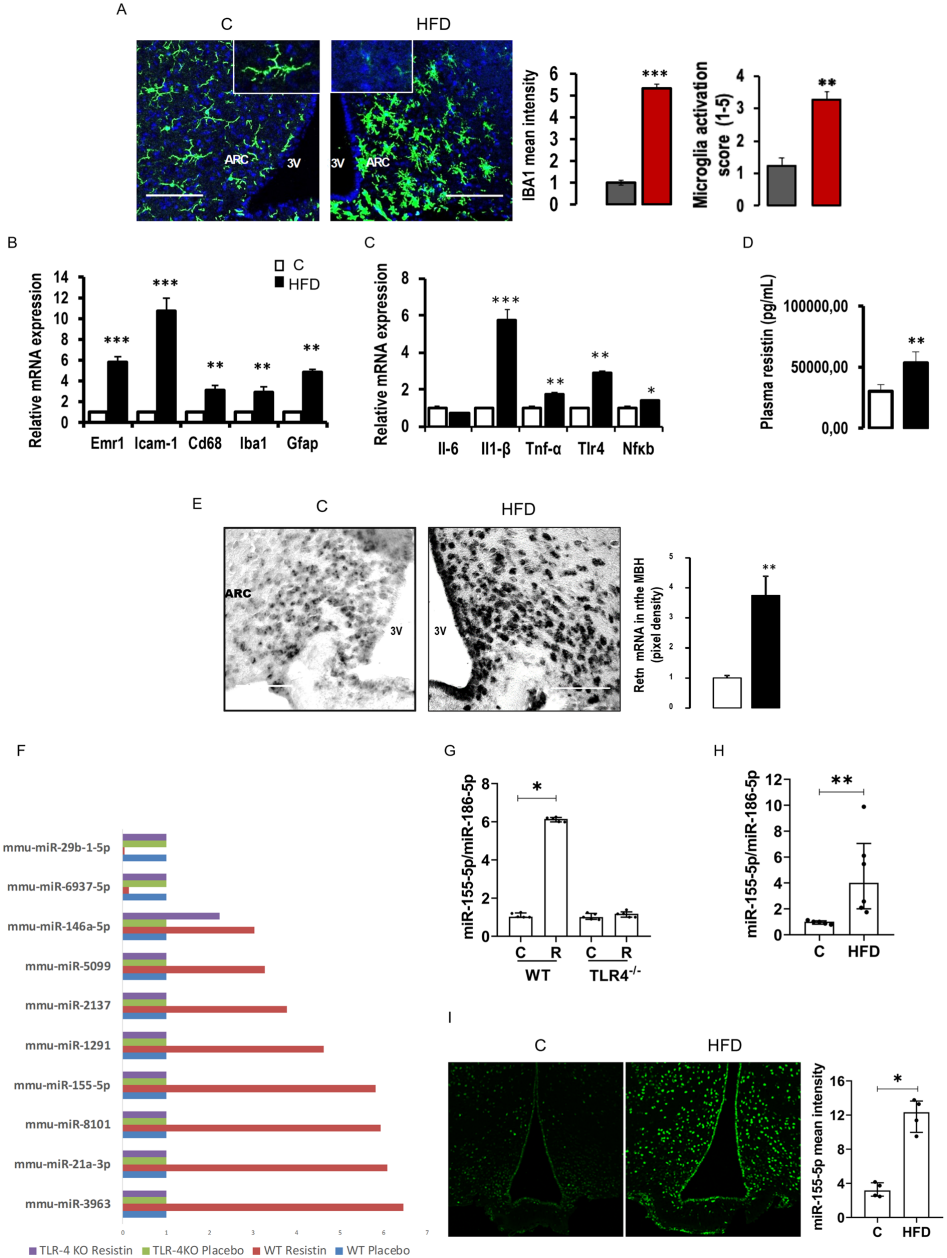
High-fat diet consumption increases hypothalamic inflammation, resistin expression, and miR-155-5p expression. (**A**) Immunohistochemical detections of Iba1 in the ARC of mice fed with chow (**C**) or HFD for 8 weeks (HFD). The rectangles integrated to Iba1 staining images represent higher magnification views of microglia under chow (left) or HFD (right) conditions in order to show microglia’s shape, in the right, quantification of Iba1 mean intensity and microglia activation score. (**B** and **C**) Quantification by RT-qPCR of pro-inflammatory markers (B) and microgliosis and astrogliosis markers in the hypothalamus of C and HFD mice. (**D**) Measurement by ELISA of resistin plasma levels in C and HFD mice. (**E**) ISH detection of resistin expression in the ARC of mice fed with chow diet or HFD for 8 weeks, and the quantification of resistin signal intensity is presented below (**E**). (**F**) Selection of microRNAs identified in the miRnome of wild type or TLR4^−/−^ (TLR-4KO) mice treated by placebo or resistin for three days through ICV route, results are normalized to placebo that represents 1. (**G**) Quantification by RT-qPCR of miR-155-5p in the hypothalamus of wild type (WT) and TLR4^−/−^ (TLR-4KO) mice in response to three days resistin or placebo ICV treatment. (**H**) Quantification of miR-155-5p expression in the hypothalamus of mice fed Chow diet or HFD. (**I**) ISH detection of miR-155-5p expression in the hypothalamus of mice fed with Chow diet or HFD for 3 days, and the quantification of miR-155-5p signal intensity is presented in the right panel. Scale bar = 50 μm (**A**). Data are means ± SEM (*n* = 3–6/group). Significant difference at *P < 0.05 **P < 0,001, and *** *P* < 0,001 versus chow-fed controls; or resistin vs. placebo

To investigate the impact of a high-fat diet (HFD) on hypothalamic inflammation and whole-body insulin sensitivity, 8-week-old male C57BL/6J mice were fed either a normal Chow diet (C) or an HFD for 8 weeks. Mice fed HFD showed greater body weight gain, increased fat mass, and higher energy intake as compared to the C group (Fig. S1 A-C). We confirm that HFD reduced plasma levels of adiponectin while increasing those of leptin and insulin (Fig S1D, E, and F). Additionally, HFD elevated resistin plasma levels (Fig. 1D). Furthermore, HFD-fed mice exhibited both glucose and insulin intolerance compared to control (C) mice (Fig. 1S G and H). HFD consumption also increased the expression of SOCS3, a negative regulator of insulin signaling, in the liver (Fig. S1 I), muscle (Fig. S1 J), adipose tissue (Fig. S1K) and hypothalamus (Fig. S1 O), whereas PTP-1B expression remained unaffected. Moreover, HFD was associated with increased gene expression of IL6, TNFα, IL1β, and TLR4 in liver (Fig. S1 L) and adipose tissue (Fig. S1 N), as well as increased expression of IL1β, and TLR4 in the muscle (Fig. S1 M). In the hypothalamus, HFD mice exhibit increased mRNA expression of IL1β, TNFα, NFκb, and TLR4 compared to control (C) mice (Fig. 1B). To determine whether hypothalamic inflammation is associated with microglia activation, we measured IBA1 expression, a microglia marker, by assessing staining intensity in the mediobasal hypothalamus (MBH). Our results show a significant increase in fluorescent intensity in IBA1-positive cells. Microglia activation is further evidenced by changes in cell morphology and an elevated microglia activation score (Fig. 1A). Consistent with these findings, the mRNA levels of markers that reflect microglia activation (CD68, Emr1, Icam1 and Iba1) and an astrocyte-specific marker (GFAP) were also increased in the hypothalamus of HFD mice (Fig. 1C).

In addition, following HFD consumption, resistin mRNA expression was measured in the MBH. Our results show that HFD-fed mice exhibit a significant overexpression of resistin in the MBH compared to control (C) mice (Fig. 1E). This augmentation is observed in the arcuate nucleus as well as in tanycytes, cells bordering the third ventricle.

### Resistin intracerebroventricular treatment and high-fat diet (HFD) consumption increase miR-155-5p levels in the hypothalamus

We previously reported that resistin promotes hypothalamic inflammation and insulin resistance through the TLR4 receptor. These effects may result from the hypothalamic gene expression modulation in response to resistin treatment. Therefore, we investigated the impact of resistin on hypothalamic microRNA (miRNA) expression profiles that could be implicated in the onset of hypothalamic neuroinflammation.

To achieve this, wild-type and TLR4⁻/⁻ mice were infused with either a placebo or resistin via intracerebroventricular (ICV) treatment for three days. Differentially expressed miRNAs (miRs) were analyzed as described in the *Materials and Methods* section. Additionally, to verify the resistin effeciency, hypothalamic expression levels of NPY and POMC were measured. As expected, resistin treatment significantly reduced POMC expression while increasing NPY expression (Fig. S2). These effects were completely abolished in TLR4⁻/⁻ mice (Fig. S2).

Next, we analyzed the hypothalamic miRNA expression profile (miRnome) across the four experimental groups. The complete miRnome is reported in Table S1. Our analysis identified seven miRNAs that were upregulated in response to resistin ICV infusion and two that were downregulated (Fig. 1F). Notably, all differentially expressed miRNAs responded to resistin in a TLR4-dependent manner, except for miR-146a-5p, which exhibited a TLR4-independent upregulation.

Among the upregulated miRNAs, we identified miR-155-5p, which has been previously linked to peripheral inflammation. Given its potential role in neuroinflammation, we focused on miR-155-5p and validated its upregulation (approximately sixfold) in response to ICV resistin treatment compared to placebo-treated mice (Fig. 1G). The effect of resistin on miR-155-5p was completely abolished in TLR4⁻/⁻ mice, confirming that resistin induces miR-155-5p in a TLR4-dependent manner (Fig. 1G).

Since we previously demonstrated that a high-fat diet (HFD) increases resistin expression in the hypothalamus, and that resistin ICV treatment upregulates miR-155-5p, we investigated whether HFD also increases miR-155-5p expression. RT-qPCR analysis revealed a significant increase in miR-155-5p expression in the hypothalamus of HFD-fed mice, approximately fourfold compared to controls (Fig. 1H). Furthermore, fluorescence in situ hybridization (FISH) analysis showed that three days of HFD feeding increased miR-155-5p expression in the hypothalamus and cells bordering the third ventricle, corresponding to tanycytes (Fig. 1I).

### Resistin upregulates miR-155-5p expression in the microglial cell line SIMA9 in a TLR-4-dependent manner

**Fig. 2:**
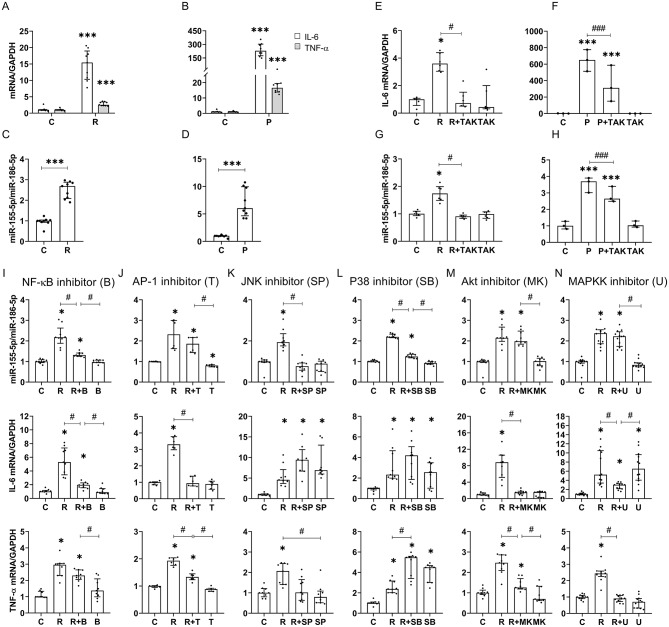
Resistin and palmitate increase miR-155-p expression in SIMA9 microglial cell line, and characterization of involved signaling pathways. SIMA9 cells were treated for 16 h in the presence of resistin (**A** and **C**) or palmitate (B and D) and then IL6 and TNFα (**A**, **B**) and miR-155-5p (**C**, **D**) expression were measure by RT-qPCR. SIMA9 cells were treated for 16 h in the presence of resistin (**E** and **G**) or palmitate (**F** and **H**) in the presence or absence of TAK and then IL6 (**E**, **F**) and miR-155-5p (**G**, **H**) expression were measure by RT-qPCR. SIMA9 cells were treated for 16 h with resistin in the presence of inhibitors of NF-kB (**I**), AP1 (**J**), JNK (**K**), p38 (**L**), Akt (**M**) or MAPKK (**N**), and then miR-155-5p, IL6 and TNFα expressions were measure by RT-qPCR. Data are median with interquartile range (*n* = 3 independent experiments and each experiment was performed with 3 technical replicates). Significant difference at * or ^#^*P* < 0.05 ** or ^##^*P* < 0,005 and *** or ^###^*P* < 0,001. * Comparison to control, and # indicates differences between other conditions

To elucidate the mechanisms by which resistin and palmitate (used to mimic the effects of a high-fat diet) induce hypothalamic miR-155-5p expression, we utilized the SIM-A9 mouse microglia cell line. Our findings demonstrated that both resistin and palmitate upregulate the expression of pro-inflammatory cytokines IL-6 and TNF-α (Fig. 2A and B) and miR-155-5p (Fig. 2C and D).

To determine whether resistin or palmitate regulates miR-155-5p expression in other neural cells, we examined the mHypo neuronal cell line and primary cultured mouse astrocytes. Resistin treatment did not affect miR-155-5p expression in mHypo cells (Fig. S3A) nor astrocytes (Fig. S3C). In contrast, palmitate induced a slight but significant increase in miR-155-5p expression in mHypo cells (Fig. S3B), though no such effect was observed in astrocytes (Fig. S3D).

Furthermore, we demonstrated that TAK, a TLR4 inhibitor, completely abolished resistin-induced IL-6 (Fig. 2E) and miR-155-5p (Fig. 2G) expression. However, TAK did not affect palmitate-induced IL-6 (Fig. 2F) or miR-155-5p (Fig. 2H), indicating that resistin’s effects are TLR4-dependent, whereas palmitate acts through a TLR4-independent pathway.

### Implicated signaling pathways in resistin-induced miR-155-5p in SIMA9 microglia cell line

Next, we investigated the signaling pathways that modulate resistin-induced miR-155-5p expression and pro-inflammatory cytokine production in SIM-A9 cells. To this end, we employed selective inhibitors targeting key pathways implicated in neuroinflammation, including NF-κB, AP-1, JNK, p38 MAP kinase, Akt, and MAP kinase. Following stimulation of SIM-A9 cells with resistin, in the presence or absence of these inhibitors, we measured the expression levels of miR-155-5p and the pro-inflammatory cytokines IL-6 and TNF-α.

Inhibition of NF-κB significantly reduced resistin-induced miR-155-5p and IL-6 expression but had no effect on TNF-α levels (Fig. 2I). Blocking JNK completely abolished resistin-induced miR-155-5p expression and reduced TNF-α levels, but did not affect IL-6 expression (Fig. 2K). In contrast, inhibition of p38 MAP kinase significantly diminished resistin-induced miR-155-5p expression without impacting TNF-α or IL-6 levels (Fig. 2L).

Interestingly, inhibition of AP-1 (Fig. 2J), Akt (Fig. 2M), or MAPKK (Fig. 2N) did not alter resistin-induced miR-155-5p expression but reduced IL-6 production and, to a lesser extent, TNF-α levels. Similar results were observed when Akt signaling was specifically inhibited.

### Characterization of miR-155-5p targets in SIMA9 microglial cells and in the whole male and female hypothalamus

**Fig. 3:**
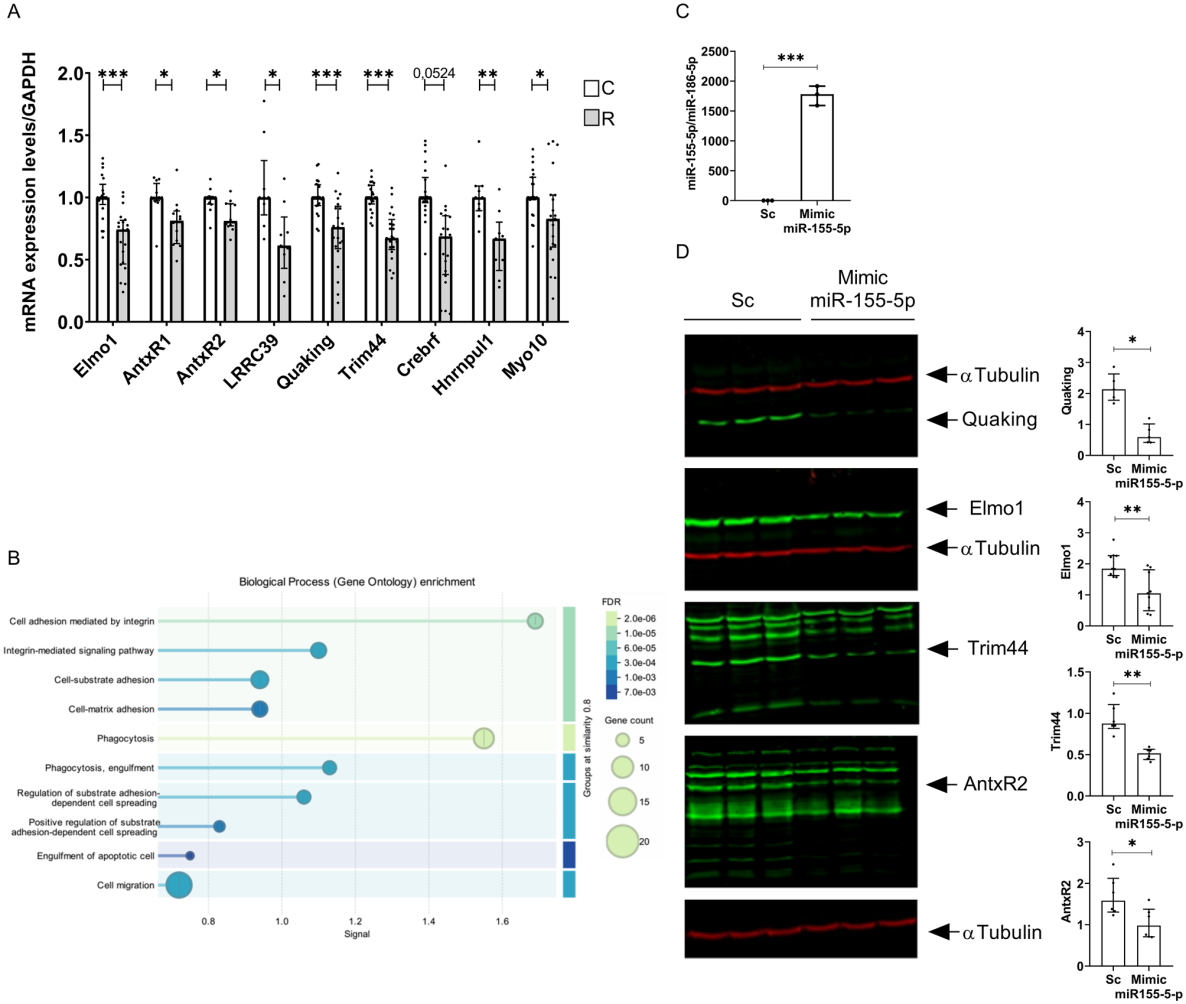
Validation of miR-155-5p targets in SIMA9 microglial cell line. (**A**) SIMA9 cells were treated with placebo (**C**) or resistin (**R**) for 16 h and then the expression of 9 miR-155-5p targets identified by HITS-CLIP were measured by RT-qPCR. (**B**) Schema showing biological process of the identified and validated miR-155-5p targets using STRING 2.0 software. (**C**) SIMA9 were transfected with miR-155-5p mimic or scrambled sequences and then miR-155-5p expression was measured by RT-qPCR. (**D**) SIMA9 were transfected with miR-155-5p mimic or scrambled sequences, then Western blot analysis was performed to identify four selected miR-155-5p targets, the quantification using LICOR software is presented on the right of the blots. Data are median with interquartile range (*n* = 3 independent experiments and each experiment was performed with 3 technical replicates). Significant difference at * *P* < 0.05, ** *P* < 0,005 and *** *P* < 0,001

Since we have demonstrated that both palmitate and resistin significantly increase the expression of miR-155-5p in SIM-A9 cells, we adapted the HITS-CLIP technique to identify miR-155-5p targets. The resulting chimeras, composed of miR-155-5p and target mRNAs, were sequenced to identify miR-155-5p targets. We identified 19 miR-155-5p targets in SIM-A9 cells (Table S2). These targets were subsequently validated through RT-PCR, confirming the downregulation of 9 targets in response to resistin treatment (Fig. 3A). Gene Ontology (GO) enrichment analysis, performed using STRING software (Version 12.0), indicated that these targets are primarily involved in processes such as phagocytosis, engulfment, endocytosis, and cytoskeleton organization (Fig. 3B).

To further validate the identified miR-155-5p targets, we examined the effect of the miR-155-5p mimic on the protein expression levels of Qk, Elmo1, Trim44, and AntxR2. First, we demonstrated that the miR-155-5p mimic increases miR-155-5p expression in SIM-A9 cells by approximately 1600-fold (Fig. 3C). Western blot analysis revealed a significant decrease in the protein expression of Qk, Elmo1, Trim44, and AntxR2 (Fig. 3D).

Additionally, we identified miR-155-5p targets in the entire hypothalamus of adult male and female mice using the HITS-CLIP technique. The list of identified targets is provided in Table S3. Our findings include targets similar to those identified in SIM-A9 cells, as well as others, since the analysis encompasses the entire hypothalamus. Using STRING software to analyze these targets, we observed in both males and females that the associated biological processes to miR-155-5p targets are primarily involved in cytoskeleton regulation, cell adhesion, and integrin signaling (Fig. S4).

### The knock-down of hypothalamic miR-155-5p attenuates HFD-induced glucose intolerance in mice

Since HFD-induced hypothalamic neuroinflammation triggers alterations of glucose tolerance, we investigated the role of miR-155-5p. Following stereotaxic injection of an AAV8-cre into the arcuate nucleus of adult male and female miR-155-5p^loxP/loxP^ mice, the mice were subjected to Chow or HFD diet for three days. The knockdown of miR-155-5p was validated in *miR-155-5*^*^loxP/loxP*^ mice treated with AAV8-Cre compared to control-treated mice, as shown in Figure S5A and S5B. Additionally, preliminary data demonstrate the co-localization of miR-155-5p and the microglial marker IBA1 using a combination of miRscope and RNAscope to label miR-155-5p and microglia, respectively (Figure S5C).

**Fig. 4:**
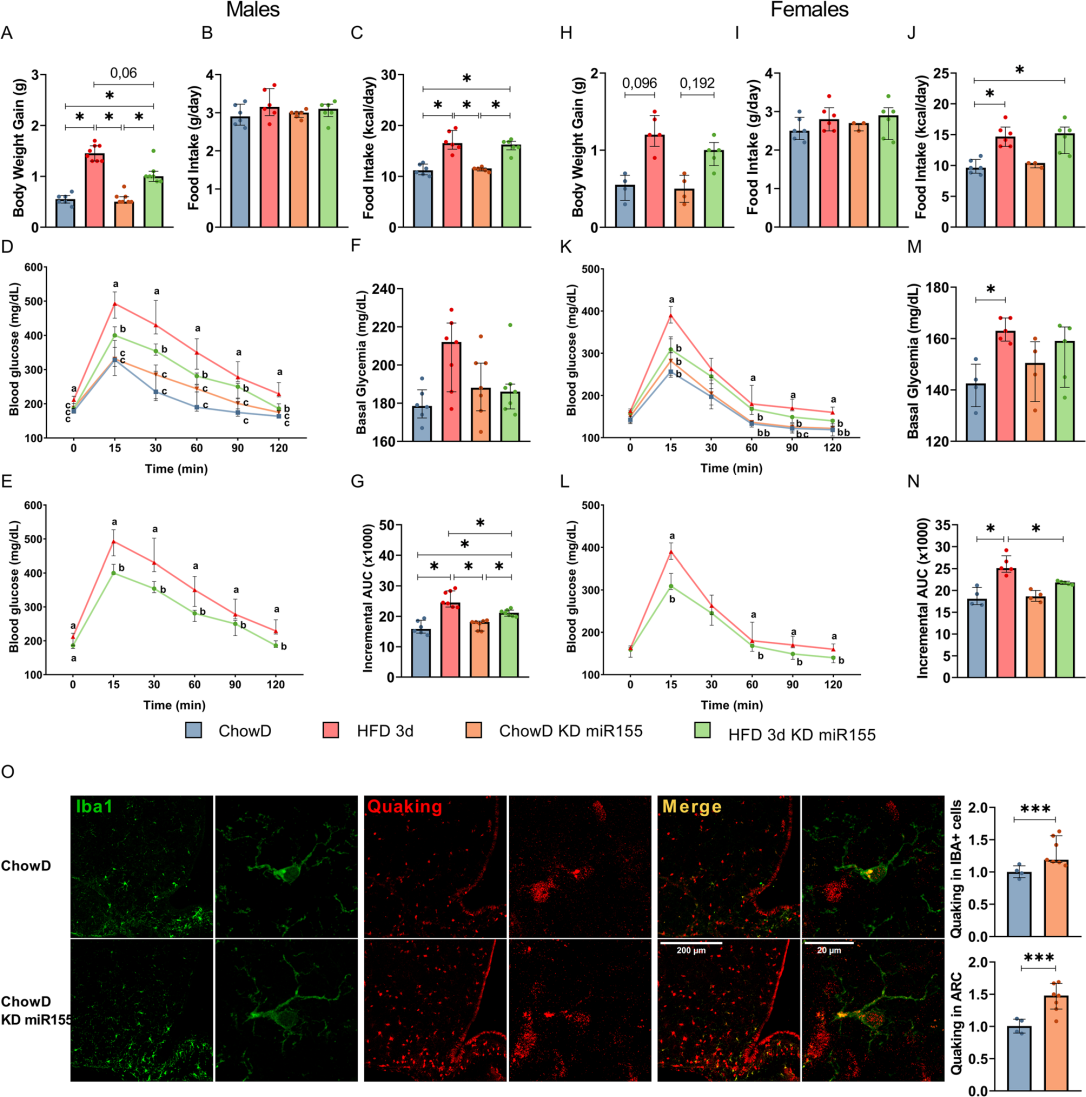
The knockdown of miR-155-5p in the arcuate nucleus improves glucose tolerance and upregulates QK. Adult male and female miR-155-5p^loxP/loxP^ mice (8 weeks of age) were treated with a single injection of AAV8-Cre or AAV8 bilaterally in the arcuate nucleus, and then fed for 3 days with Chow or HFD diets. Basal glycemia, body weight gain, food intake (g/day), food intake (kcal/day) and basal glycemia were measured in males as shown in **A**, **B**, **C**, and **F** panels, respectively; and in females as shown respectively in H, I, J and M panels. The glucose tolerance is presented for the four groups in males (panels **D** and **E**), and the AUC is presented in panel **G**, and in females (panels **K** and **L**), and the AUC is presented in panel **N**. (**O**) IHC was performed in control and miR-155-5p knockdown mice fed a Chow diet. IBA1 and QK-specific stainings were measured using specific antibodies. QK expression was measured in IBA1-positive cells and reported in the histogram below the IHC images. Data are median with interquartile range (*n* = 5–8). Significant difference at * *P* < 0.05, and *** *P* < 0,001. For the glucose tolerance test, different letters indicate *p* < 0.05

Knockdown of miR-155-5p did not affect body weight gain in response to an HFD in female mice (Fig. 4H). In male mice, HFD increased body weight gain in both control and miR-155-5p knockdown groups (Fig. 4A), the increase was less pronounced in miR-155-5p knockdown mice (Fig. 4A). Basal glycemia was unaffected by either HFD or miR-155-5p knockdown in male mice (Fig. 4F). In contrast, HFD increased plasma glucose levels in female mice when comparing Chow diet to HFD, but this effect was not observed in the knockdown group (Fig. 4M). Additionally, HFD increases energy intake independently of miR-155-5p knockdown in male (Fig. 4C) and female (Fig. 4J) mice. Furthermore, in control mice, HFD consumption for three days increases glucose intolerance in male (Fig. 4D, E) and female (Fig. 4K, L) mice. However, miR-155-5p knockdown in the arcuate nucleus significantly reduced HFD-induced glucose intolerance in both male (Fig. 4D, D) and female (Fig. 4K, L) mice, as also showed by the analysis of incremental AUC for male and female, Fig. 4G and N, respectively.

### The knock-down of hypothalamic miR-155-5p increases quaking expression in the hypothalamic microglia cells

Qk is amongst miR-155-5p targets identified by HITS-CLIP in the hypothalamus and SIMA9 microglia cell line. We investigated the impact of miR-155-5p knockdown on QK expression in hypothalamic microglia. Following, stereotaxic injection of an AAV8-cre into the arcuate nucleus of adult male and female miR-155-5p^loxP/loxP^ mice, the mice were subjected to three days of a Chow diet. We colocalized QK, using specific Qk antibodies, with microglia, using IBA1 antibodies. We show that miR-155-55p knockdown significantly increases the expression of Qk in microglia (Fig. 4M).

## Discussion

The hypothalamus controls energy homeostasis by integrating hormonal and neuronal signals. Chronic low-grade inflammation in this region, triggered by hypercaloric diets such as a high-fat diet (HFD), is called hypothalamic neuroinflammation and has been strongly associated with metabolic disorders, including obesity and type 2 diabetes. This neuroinflammatory process is characterized by microglial activation and increased expression of pro-inflammatory cytokines, which can disrupt neuronal signaling pathways involved in metabolic regulation.

Our study confirmed that HFD induces hypothalamic neuroinflammation, as evidenced by microglial activation and increased pro-inflammatory cytokine expression. These effects are likely mediated by systemic inflammation and alterations in circulating adipokines, particularly resistin. Resistin has been primarily implicated in insulin resistance and metabolic disorders; however, emerging evidence suggests its role in neuroinflammation, especially in the hypothalamus. Central administration of resistin has been shown to modulate hypothalamic neuronal activity, impair liver insulin responsiveness, and regulate glucose homeostasis [11, 24]. Despite these findings, the specific effects of HFD on hypothalamic resistin expression remain poorly explored.

Our findings demonstrate that HFD increases hypothalamic resistin expression, consistent with prior studies identifying resistin immunoreactivity in POMC-positive cells [25]. Interestingly, resistin expression was not detected in astrocytes or microglia, suggesting that its involvement in HFD-induced hypothalamic neuroinflammation may be indirect. One potential mechanism linking resistin to neuroinflammation involves the nuclear factor kappa B (NF-κB) pathway, a key regulator of inflammatory responses. Resistin has been shown to activate NF-κB in microglia, and its intracerebroventricular (ICV) administration promotes neuronal inflammation while modulating insulin, adiponectin, and FGF21 signaling in a TLR4-dependent manner [12, 13].

Additionally, resistin may regulate hypothalamic gene expression through microRNAs (miRNAs), which influence mRNA translation and protein synthesis. Our study identified miR-155-5p as a key downstream target of resistin in the hypothalamus, with resistin-induced miR-155-5p expression occurring in a TLR4-dependent manner. MiR-155 has been widely implicated in immune cell inflammation [26–28] and contributes to the p53-mediated pro-inflammatory network in microglia [29]. Our results show that HFD upregulates hypothalamic miR-155-5p expression, likely mediated by increased hypothalamic resistin levels. Using the SIMA9 microglial cell line, to more investigate this pathway, we demonstrate that resistin enhances pro-inflammatory cytokine production and miR-155-5p expression in microglia. Similar effects were observed following palmitate treatment, although the underlying mechanisms differed. Notably, the resistin-induced upregulation of miR-155-5p and IL-6 was abolished by the TLR4 inhibitor TAK, whereas palmitate-induced effects were only mildly affected, suggesting that palmitate may act through an alternative, TLR4-independent pathway. These findings align with reports that question TLR4’s role as a receptor for palmitate [30, 31], although some studies suggest that palmitate may exert effects through direct TLR4 binding [32, 33].

Interestingly, neither resistin nor palmitate increased miR-155-5p expression in primary cultured astrocytes. However, palmitate, but not resistin, elevated miR-155-5p expression in the mHypo neuronal cell line, suggesting that microglial cells are the primary targets of resistin-induced miR-155-5p expression in the hypothalamus. This observation underscores the cell-type specificity of inflammatory responses to metabolic insults and highlights the complex interplay between different hypothalamic cell populations in mediating neuroinflammatory processes.

We utilized inhibitors of major pro-inflammatory pathways to delineate the signaling pathways linking resistin to miR-155-5p expression. Inhibition of NF-κB, JNK, or p38 MAP kinase significantly reduced resistin-induced miR-155-5p expression. Given that the promoter region of the mouse Bic/Mir155 gene contains binding sites for transcription factors such as AP-1 and NF-κB [34, 35], our findings suggest that resistin modulates miR-155-5p expression via TLR4 signaling pathways involving JNK, NF-κB, and p38 MAP kinase. Interestingly, while AP-1, Akt, and MAPKK inhibitors abolished resistin-induced IL-6 expression, they did not affect miR-155-5p expression, indicating that not all resistin-mediated inflammatory effects are miR-155-5p-dependent.

Our findings underline the resistin/TLR4/miR-155-5p axis as a critical contributor to hypothalamic neuroinflammation, particularly through microglial activation. Using HITS-CLIP, we identified miR-155-5p targets in SIMA9 cells and hypothalamic tissue, which were predominantly involved in phagocytosis, engulfment, and endocytosis—processes closely linked to microglial activation. Notably, in both males and females, miR-155-5p targets are involved in cell adhesion, integrin signaling, and cell motility. We also observed sex-specific differences in miR-155-5p targets, as shown in Fig. 4S. This finding aligns well with previously published results reporting sex-dependent microglial activation in the context of energy homeostasis, involving the CX3CR1 signaling pathway [36]. Among the common targets across SIMA9 cells and hypothalamic samples were Qk, Elmo1, AntxR2, Trim44, and Hnrnpul1, proteins primarily involved in cytoskeleton organization and inflammatory responses.

ELMO1, a key regulator of neuroinflammation, facilitates immune cell migration and inflammatory signaling by interacting with DOCK180 to activate Rac1, a small GTPase that modulates the actin cytoskeleton [37]. Additionally, TRIM44 enhances autophagy in response to oxidative stress by promoting SQSTM1 (p62) oligomerization [38]. ANTXR2, another identified target, has been implicated in neuronal signaling, extracellular matrix regulation, and autoimmune neuroinflammatory diseases [39]. Given its central role in glial cell function, we focused on QKI, an RNA-binding protein crucial for neuroinflammation, as its dysregulation has been linked to demyelinating diseases [40, 41]. In SIMA9 cells, miR-155-5p mimics led to QKI downregulation, while in vivo knockdown of miR-155-5p in the mediobasal hypothalamus restored QKI expression and improved glucose tolerance. The underlying mechanisms by which hypothalamic QKI may regulate glucose homeostasis remain unclear and require further investigation. However, at the peripheral level—particularly in adipose tissue—QKI has been implicated in the regulation of metabolism and thermogenesis [42].

These findings reinforce the pathological role of the resistin/TLR4/miR-155-5p pathway in neuroinflammation and metabolic dysfunction. Future studies should explore therapeutic strategies targeting this axis to mitigate HFD-induced hypothalamic inflammation and its metabolic consequences. Investigating the long-term effects of resistin inhibition on neuroinflammatory markers and metabolic parameters could provide valuable insights into its potential as a clinical intervention for obesity-associated disorders. Furthermore, elucidating the role of additional miRNAs in resistin-mediated neuroinflammation may reveal broader regulatory networks that contribute to hypothalamic dysfunction in metabolic disease.

## Conclusions

Our study provides novel insights into the mechanisms underlying hypothalamic neuroinflammation. We demonstrate that HFD increases hypothalamic resistin expression, which increases the expression of miR-155-5p in microglia, leading to the downregulation of key targets involved in neuroinflammatory responses. Importantly, inhibition of this pathway improves glucose homeostasis, highlighting its potential as a therapeutic target for metabolic disorders associated with neuroinflammation.

## Materials and methods

### Animals

Adult male C57BL/6J mice (Janvier Labs, Le Genest-St-Isle, France), TLR4 knockout (TLR4^−/−^) mice (TAAM-UPS44; CNRS, Orleans, France), and miR-155-5p^loxP/loxP^ 8-week-old male and female mice were housed under specific pathogen-free conditions in a temperature-controlled environment(21–22 °C) with a 12-h light/dark cycle and had free access to water and food. Mice were fed with standard chow diet (19,6% protein, 11,9% lipids, 68,5% carbohydrates) or a high-fat diet (HFD 235, 15,6% proteins, 44,6% lipids, 39,9% carbohydrates; Safe, Augy, France) for 3 days, 8 days or 8 weeks (LT-HFD). Animals weight and food intake were regularly measured (daily for ST-HFD, and every 2–3 days for LT-HFD). miR-155-5p^loxP/loxP^ mice were fed with standard chow diet (D12450J, 20% protein, 10% fat, 70% carbohydrates) or a high-fat diet (D12492. 20% protein, 60% fat, 20% carbohydrates) (from Research Diet). All experimental procedures were performed according to the institutional guidelines for animal use specified by the European Union 621 Council Directive (2010/63/EU), and approved by the French Ethics Committees for the Care and Use of Experimental Animals (C2EA, 59 Comity Paris Centre et Sud; authorization n° 27899 and authorization n° 43178). At the end of the experiment period, glucose and/or insulin tolerance tests were assessed. Then, tissues were manually collected and immediately frozen into liquid nitrogen and conserved at -80 °C until use. Brains were fixed overnight into 4% Paraformaldehyde/PBS solution and then placed into 10% sucrose/PBS for 24 h and then in 20% sucrose/PBS solution for 24 h. Brains were then frozen in -40 °C cooled solution of isopentane and then conserved at -80 °C.

### Intracerebroventricular (ICV) infusion of resistin

Adult male C57BL/6J mice and TLR-4 knockout mice were ICV injected with either resistin (Shenandoah Biotechnology, Warwick, USA) or vehicle (saline) as previously described (Benomar et al., 2016). Briefly, mice were anesthetized with 0.5–2.0% isoflurane in 95% O_2_ / 5% CO_2_ mixture and placed in a stereotaxic frame. The brain infusion cannulas were stereotaxically placed into the lateral brain ventricle using the following coordinates 0.58 mm anterior to bregma, 1 mm lateral, and 2 mm dorso-ventral. The osmotic pumps (model 1003; Alzet, Charles River, L’Arbresle, France) were housed in a subcutaneous pocket on the dorsal surface of the animal. C57BL/6J and TLR4-KO mice were infused for 3 days with vehicle or resistin (2 µg/24µL/day; pumping rate 1µL/h). At the end of the experimental period, tissues samples were collected, snap frozen in liquid nitrogen and stored at -80 °C until use. For immunohistochemical analyses, brains were collected from mice transcardially perfused with 4% PFA, and processed for immunofluorescence and confocal analyses (6–8 animals/group).

### Knock-down of miR-155-5p

Adult male and female miR-155-5p^loxP/loxP^ mice were treated with a single injection of AAV8-Cre bilaterally in the arcuate nucleus. After three weeks, mice were subjected to Chow or HFD for three days. At the end of the experimental period, brains were collected from mice transcardially perfused with 4% PFA, and processed for immunofluorescence and confocal analyses (6–8 animals/group).

### Cell culture

We have used SIM-A9 (spontaneously immortalized murine microglia, ATCC^®^ CRL-3265 cells). Cells were maintained in Dulbecco’s modified Eagle’s medium high glucose (DMEM 4,5 g/L) mixed 1:1 with Ham’s F-12 supplemented with 10% Fetal bovine serum, 5% Horse serum, 1% Penicillin-streptomycin, 1% L-Glutamine (all Gibco; Thermo Fisher Scientific) in a 5% CO_2_ atmosphere at 37 °C. The mouse hypothalamic cell line mHypo (purchased from Cellutions Biosystem Inc.) were maintained in Dulbecco’s modified Eagle’s medium high glucose (DMEM 4,5 g/L) supplemented with 10% Fetal bovine serum, 1% Penicillin-streptomycin, 1% L-Glutamine (all Gibco; Thermo Fisher Scientific) in a 5% CO_2_ atmosphere at 37 °C.

### Cell stimulation

Serum starved SIMA9 cells were treated in the presence or absence of resistin (75 ng/mL), 0.5 mg/mL lipopolysaccharide (LPS) or 0.5 mM Palmitate (Pal). The palmitic acid was dissolved in fatty acid-free bovine serum albumin (BSA 13.2%) solution. In another set of experiments, we aimed to investigate the involved signaling pathways we have used specific inhibitors of key pathways. Thus cells were treated with: 20 µM SP-600,125 (Sc 200636, Santa Cruz, USA) an inhibitor of JNK pathway, 10 µM U-0126 (Sc 222395, Santa Cruz, USA) an inhibitor of MAPKKinase pathway, 1 µM MK-2206 dihydrochloride (Sc 364537, Santa Cruz, USA) an inhibitor of AKT pathway, 50 µM SB-202,190 (Sc 222294, Santa Cruz, USA) an inhibitor of p38 pathway, 1 µM TAK-242 (243984-11-4, Calbiochem from Merck, Germany) and inhibitor of TLR4, 10 µM Tanshinone IIA (BML-GR 336, Enzo Life, Villeurbanne, France) an inhibitor of AP-1 pathway, or 5 µM BAY 11-7082 (BML-EI278-0010, Enzo Life, Villeurbanne, France) an inhibitor of NF-κB pathway.

### RNA extraction from mice tissues or cells

Total RNA was extracted from frozen tissues samples using a TRIzol™ reagent from Invitrogen (Illkirch, France) according to the manufacturer’s instruction. Tissues were then homogenized using a PreCellys homogenizer (PreCellys 24/Cryolys) for 30s at 4 °C. For cells total RNA was extracted using TRIzol™ reagent from Invitrogen (Illkirch, France) according to manufacturer’s recommendations.

### Reverse transcription (RT) and quantitative real-time polymerase chain reaction (qPCR)

RNA concentration and purity were measured using a NanoDrop ND1000 spectrophotometer (ThermoFisher Scientific, Courtaboeuf, France).

A total of 1 µg of RNA was reverse transcribed (RT) using qScript™ cDNA Synthesis Kit from VWR (Fontenay sous Bois, France), according to the manufacturer’s instructions. Real Time quantitative polymerase chain reaction (qPCR) analysis was performed using a SYBR Green PCR Master Mix kit from VWR (Fontenay sous Bois, France) with an CFX96™ Real-Time System C1000 Touch Thermocycler (Bio-Rad, les Ulis, France) using specific primer pairs. All primers were purchased from Eurofins (Köln, Germany), all primer pairs are listed in table S4.

### Measurement of MicroRNAs expression

Total RNAs were used for the quantification of miRNA expression using TaqMan Advanced MicroRNA Assays (ThermoFisher, Courtaboeuf, France), according to the manufacturer’s instructions. In brief, TaqMan™ Advanced miRNA cDNA Synthesis Kit was used for retro-transcription. This technique consisting of an RNA-specific stem-looped primer for the reverse transcription and forward primers, reverse primers and FAM™ dye-labeled MGB probe for the qPCR. Thus, RT-qPCR was performed using TaqMan Fast Advanced Master Mix (ThermoFisher, Courtaboeuf, France) and specific TaqMan assays.

The expression levels of miRNA in each sample were measured in terms of threshold cycle (Ct) value and normalized to hsa-miR-26b-5p or hsa-miR-186-5p, which was used as an internal control. PCR cycles were as follows: 3 min pretreatment at 95 °C, 95 °C for 10 s and 60 °C for 30 Sect. (40 cycles).

Reverse transcription and qPCR reagents were purchased from Applied Biosystems (Thermo Fisher Scientific, Courtaboeuf, France).

GAPDH and β-actin were used as controls of the input RNA and miR-186-5p for miRNA level. All samples were measured in duplicates. Relative gene expression was determined using the CFX manager software (Bio-Rad CFX Manager 3.1) with the 2^−ΔΔCq^ method. Raw Cq value > 35 was considered as undetectable.

The TaqMan^®^ primers were designed from Applied Biosystems: mature miR-26b-5p sequence: UUCAAGUAAUUCAGGAUAGGU, mature miR-155-5p sequence: UUAAUGCUAAUUGUGAUAGGGGU and mature miR-186-5p sequence: CAAAGAAUUCUCCUUUUGGGCU.

### MiRnome cDNA library construction

Total RNA (2 µg) extracted from the hypothalamus of mice treated by placebo or ICV resistin for 3 days were added to an equal volume of formamide, heated at 70 °C for 3 min, and loaded on a denaturing urea (8 M) polyacrylamide (17%) gel for size-fractionation. In all cases, the high quality of RNAs was checked by a lack of any smear and the fact that the tRNAs, 5 S RNA and 5.8 S RNA migrated as discrete bands. Small RNAs of 18–36 nucleotides were eluted from the corresponding slices by overnight incubation in NaCl 0.4 M (0.8 ml) at 4 °C under gentle shaking. Eluates were precipitated by adding 2.5 volumes of ethanol in the presence of glycogen (10 ng), rinsed twice with ethanol 70%, and resuspended in RNase-free H_2_O (10 µl).

Individual cDNA libraries were built following an Illumina-like protocol in which 3′- and 5′-adapters were sequentially ligated at the 3′- and 5′-ends of small RNAs, respectively, to allow for their reverse transcription (RT) and amplification by polymerase chain reaction (PCR) as previously described [43]. The 3′-Adaptor was first adenylated by using 25 pmoles of oligonucleotides, 2 × Quick Ligation reaction buffer (New England Biolabs, Evry, France), and 1,600 U of T4 DNA ligase (New England Biolabs, Evry, France), in a volume of 50 µl and by overnight incubation at 37 °C. The adenylated and non-adenylated adaptors were size-fractioned on a denaturing urea (8 M) polyacrylamide (20%) gel. The adenylated adaptor was eluted as described above. Adenylated 3′-adaptor (0.5 µl at 0.25 µM) was then added to small RNAs (5 µl) in 0.2 ml Thermo-Tubes (Thermo Scientific, Courtaboeuf, France). To prevent secondary structures, mixes were heated for 3 min at 70 °C, then kept on ice while adding PEG 8000 (2.4 µl, 50%), truncated T4 RNA ligase 2 (0.4 µl from 200 U/µL), and 10X truncated T4 RNA ligase2 buffer (all provided by New England Biolabs) (1.0 µl). Mixes were incubated for 90 min at 25 °C. After the addition of [1 µM] 5′-adaptor (0.5 µl) and a new 70 °C/ice cycle, mixes were added with [20 U/µl] T4 RNA ligase 1 (0.75 µl) and [10 µM] ATP (1 µl) and incubated for 90 min at 25 °C. In a third step, mixes were added with [100 µM] RT-primer (0.5 µl), submitted to a 70 °C/ice cycle, then added with [0.1 M] DTT (1 µl), [10 µM each] dNTP (1 µl), 10X buffer (4 µl), and [200 U/µl] of Superscript III reverse transcriptase (0.65 µl) (ThermoFischer Scientific, Courtaboeuf, France) and incubated for 90 min at 50 °C. Finally, mixes were added with [100 µM] 3′-PCR-primer (0.6 µl), [100 µM] 5′-PCR-primer (0.3 µl), and 2X Master Mix Phusion enzyme (15 µl) (New England Biolabs). Reaction mixes were split into two tubes to enhance thermic exchange, denaturated for 1 min at 98 °C, and submitted to 16 cycles of 20 s at 98 °C, 30 s at 55 °C, 25 s at 72 °C. PCR products were size-fractionated on a 6% polyacrylamide gel so that ~ 100-bp cDNAs could be separated from the 75-bp by products corresponding to primer dimers. We used a set of 3′-PCR-primers with whole complementarity to the 3′-adaptor but a bulge of two nucleotides at position 22 that were showed not to introduce any bias in control cDNA libraries. cDNA libraries built from biological replicates were barcoded with the same 3′-PCR-primer and sequenced in different lanes of an Illumina’s genome analyzer GAIIX [44].

### MiRNA expression profiling

Sequencing quality was ascertained using the FASTQC program (http://www.bioinformatics.babraham.ac.uk/projects/fastqc*).* cDNA libraries were demultiplexed using our scripts on the basis of the first 11 nucleotides of the 3′ adaptor and reads were trimmed from the 3′ adaptor sequence. Sequencing reads < 15 nucleotides which could not be mapped on the genome were discarded. Duplicate reads > 15 nucleotides were collapsed into unique sequences and analyzed with the sRNAbench tool on the sRNAtoolbox server (http://bioinfo5.ugr.es/srnatoolbox) to build individual miRNA expression profiles [45]. This server used the miRBase database version 21 (http://www.mirbase.org) to identify miRNA sequences [46]. miRNA expression profiles were normalized using the Edger procedure [47]. Individual group statistics were calculated using Mann and Whitney tests and corrected for multiple testing when necessary, according to the false discovery rate method previously described [48, 49].

### Fluorescence immunohistochemistry

Mice under anesthesia were transcardially infused with 4% paraformaldehyde (PFA) solution. Brains were collected, post-fixed in PFA 4%, cryoprotected in 20% sucrose solution for 48 h and frozen in -40 °C cooled isopentane solution. Brain coronal sections (20 μm–10 μm thickness) through the hypothalamus were then subjected to standard immunohistochemistry protocol. Briefly, sections were first incubated with 50 mM NH4Cl for 20 min and blocked/permeabilized with PBS solution containing 0.1% Triton X-100, 0.2% Fish Gelatin, 2% normal donkey serum for 1 h at room temperature. The brain sections were incubated overnight at 4 °C with: goat anti-Iba1 or rabbit anti-Qk antibodies (1:2000; Cell Signaling Technology, USA) and then incubated with the appropriate fluorescent secondary antibodies (1:400; Invitrogen, Courtaboeuf, France). Nuclei were counterstained with DAPI (1/300 Sigma-Aldrich, USA). Images were acquired using a Zeiss LSM-700 or confocal microscope with AiryScan module with Zen software and reconstructed in Fiji Software. Quantification of fluorescence intensity was performed in a blinded fashion using ImageJ software. Both sides of the bilateral structures of the ARC were quantified, and replicate values from each animal were individually averaged.

### In situ hybridization (ISH) of miR-155-5p

Brain tissue was cut into 10 μm slices with a cryostat. After 30 min at room temperature (RT), slices are rehydrated with 1xPBS, three times for 10 min each. Slices are permeabilized with proteinase k (5 µg/mL) 10 min at 37 °C. Wash with 1xPBS, three times 10 min each. Slides were pre-hybridized/blocked with buffer composed by 5xSSC (VWR, France), 5xDenhardt’s (Qbiogene, France), 10% Dextran-sulfate (Sigma, France), 50% Formamide (Sigma, France) and yeast RNA (200 µg/mL, Sigma, France), for 1 h at 65 °C. The DIG-labeled miRCURY LNA miRNA Detection Probes (Qiagen, Courtaboeuf, France) are added to this hybridization buffer for 1h15 at 65 °C. U6 as a positive control (1nM, Qiagen, Courtaboeuf, France), Scramble as a negative control (40nM, Qiagen, Courtaboeuf, France) and miR-155-5p (40nM, Qiagen Courtaboeuf, France). Several washes were performed for 5 min each at 70 °C: 5xSSC and 50% formamide, 5xSSC, twice 1xSSC, twice 0,2xSSC. Then washes at RT, always 5 min each: 0,2xSSC and 1xPBS. Slides were blocked in 1xPBS-0,1%Tween-20, 2% Sheep serum and 1% BSA solution for 30 min at RT. Anti-Digoxigenin-POD was diluted (1:800, Sigma, France) in 1xPBS-0,1%Tween-20, 1% Sheep serum (Sigma) and 1% BSA solution and incubated 1 h at RT. Wash with 1xPBS-0,1%Tween-20, three times 5 min each. Revelation was performed with TSA^®^ Plus Cyanine 5 (Cy5) detection kit (1:200, PerkinElmer, Courtaboeuf, France) for 10 min. Wash twice with 1xPBS-0,1%Tween-20, 10 min each and once with 1xPBS for 1 min. Cell nuclei were stained with DAPI diluted in 1xPBS (1:3000, 4′,6-diamidino-2-phenylindole, Sigma-Aldrich, USA) for 3 min. Wash with 1xPBS and twice with water. Images were captured on a Zeiss LSM-700 confocal microscope.

### MiRNAscope in situ hybridization for miR-155-5p characterization

To perform the miRNAscope, miRNAscope HD Detection Reagents were used according to the manufacturer’s protocol (ACD Bio-Techne, France. Frozen sections of 10 μm were washed, dried at 60 °C for 1 h, post-fixed and dehydrated. A blocking step with hydrogen peroxide was also incorporated to block endogenous peroxidases as HorseRadish Peroxidase/Tyramide Signal Amplification chemistry was used downstream in the amplification stage of the assay. To better access target miRNA, slides were permeabilized with the Target Retrieval incubation for 10 min at 95 °C in a steamer. Protease prehybridization step used 40 min with “Protease III” at 40 °C. Samples were stained with miR-155-5p and negative control probes design by ACD Bio-techne for 2 h at 40 °C. Then, six steps of amplifications and washes with specific buffer were performed before detection with Fast Red chromogen. Images were captured with a microscope Zeiss Imager 1 ApoTome.2. The images were converted to 8-bit grayscale, standardizing intensity values on a 0–255 scale to prepare them for thresholding operations. To reduce background noise and enhance signal-to-noise contrast, background subtraction was applied using the rolling ball algorithm (Process → Subtract Background), with a radius set between 2 and 6 pixels. This radius was adjusted individually based on the background variability of each image.

After background correction, thresholding was performed using the Default method in ImageJ (Image → Adjust → Threshold). The initial threshold values were automatically set using the Auto function to establish a baseline segmentation. These values were then manually refined for each image to optimally capture the true signal while minimizing background interference. The lower threshold was fixed at 225, while the upper threshold was adjusted individually for each image to exclude saturated signals and imaging artifacts. This selective thresholding approach allowed for consistent yet tailored segmentation of bright puncta across all samples.

The images were subsequently converted into binary masks using the Make Binary function, followed by Fill Holes to complete any partial structures and Watershed to separate adjacent or overlapping puncta. Finally, puncta were quantified using the Analyze Particles function, with size set from 0 to infinity and circularity from 0 to 1, ensuring no particle outlines were displayed. This workflow provided a consistent, unbiased method for detecting miRscope-positive puncta across all samples.

### High-throughput sequencing of RNA isolated by cross linking immunoprecipitation (HITS-CLIP)

Confluent SIMA9 microglia cells were washed with PBS, then immersed with PBS to cover the monolayer and then irradiated once at 400 mJ/cm2 followed by 200 mJ/cm2 in the UV crosslinker as previously described [50]. Cells were then lysed with lysis buffer (1XPBS/1% Igepal/0.5% sodium deoxycholate/0.1%SDS) containing Complete protease inhibitor (Roche, France). Cell lysates were treated with DNAse and then with RNaseA as previously described [48]. After clearing lysates (centrifugation 50000 g at 4°C for 20 minutes), samples were incubated in the presence of Dynal Protein G beads (Life Technologies, Courtaboeuf, France) prepared with anti-AGO for overnight at 4°C, then washed as previously described [50]. Briefly, immunoprecipitation complexes were washed three times with lysis buffer containing 5XDenhardt’s solution, twice with high-detergent buffer (1XPBS/1% Igepal/1% sodium deoxycholate/ 0.2% SDS), three times with low-salt buffer (15 mM Tris pH 7.5, 5 mM EDTA), twice with high-salt buffer (1XPBS/ 1% Igepal/ 0.5% sodium deoxycholate/ 0.1% SDS/1 M NaCl, and twice with PNK was buffer (50 mM Tris pH 7.5/10 mM MgCl2/0.5% Igepal). Beads were subjected to PNK (3’-phosphatase minus) to phosphorylate cleaved mRNA 5’-ends [51], followed by chimera ligation (miRNA-mRNAs) using T4 RNA ligase I [51]. An alkaline phosphatase treatment was performed to remove 3’-phosphate groups, followed by the ligation of a 3’linker (5’-p-TGGAATTCTCGGGTGCCAAGG-Amc7) using truncated RNA ligase 2 (New England Biolabs, Evry, France). The beads bound to the chimeras were subjected to SDS-PAGE and following nitrocellulose electro-transfer, bands with AGO-bound RNA were subjected to proteinase K and RNA extraction.

To specifically construct libraries dedicated to miR-155-5p, RT was performed using 3’linker sequence as primer followed by PCR using miR-155-5p sequence as sense primer and 3’ linker as antisense primer. Finally, nested adapters for high-throughput sequencing were added to libraries with additional PCR cycles. PCR conditions and indexed primers were reported in supplementary table. Libraries were sequenced on the Illumina Miseq 2500 platform (I2BC, CNRS/University of Paris-Saclay) with 75-nucleotide single-end reads.

### Statistical analysis

All data were analyzed using R software (version 4.4.1). To compare 2 or more than 2 conditions, two-k sample permutation test with the Monte Carlo resampling approximation was performed. For more than 2 groups, test post-hoc were performed as the pairwise test or n parcomp test.

For the analysis of the GTT, pairwise comparisons between groups at each time were done by using permutation t tests and incremental area under the curve (AUC) was analyzed with R as well.

Most of the graphs were made using GraphPad Prism version 8.0.1 for Windows.

## Electronic supplementary material

Below is the link to the electronic supplementary material.

Supplementary Material 1

## Data Availability

No datasets were generated or analysed during the current study.
