# Supplementary material for: The Resistin/TLR4/miR-155-5p axis: a novel signaling pathway in the onset of hypothalamic neuroinflammation
Source: J Neuroinflammation. 2025 Aug 4;22:198. doi: 10.1186/s12974-025-03522-3 (PMC12323067; doi:10.1186/s12974-025-03522-3)
Supplement: Supplementary file 1 — Supplementary Material 1 [file 12974_2025_3522_MOESM1_ESM.docx]

**Figure S1**. Impact of HFD on metabolic parameters and inflammation in mice fed HFD for 8 weeks. (A) Indicates energy intake as kcal/kg of Chow(C) and HFD fed mice; (B) indicates body weight gain from T0 to 8 weeks; (C) indicates white adipose tissue mass in the two groups; (D), (E) and (F) indicate plasma levels of adiponectin, leptin and insulin, respectively. (G) and (H) indicate GT and ITT, respectively. Total RNAs were prepared from different tissues of mice fed Chow or HFD and subjected to RT-PCR using specific primers as indicated in the figures. (I), (J) and (K) indicate the expression levels of SOCS3 and PTP1B in liver, muscle and adipose tissue, respectively, in Chow and HFD mice. (L), (M), (N) and (O) indicate the expression levels of pro-inflammatory factors in liver, muscle, adipose tissue and hypothalamus, respectively, in Chow and HFD mice. Data are means ± SEM (n = 6-8). Significant difference at * P<0.05, ** P<0,01 and *** P<0,001.

**Figure S2.** Resistin treatment upregulates NPY and downregulates POMC in the hypothalamus of mouse.

Quantification by RT-qPCR of NPY (A) and POMC (B) in the hypothalamus of wild type and TLR4^-/-^ (TLR4-KO) mice in response to three days resistin or placebo ICV treatment. Data are means ± SEM (n = 6). Significant difference at *** P<0,001.

Fig.S3


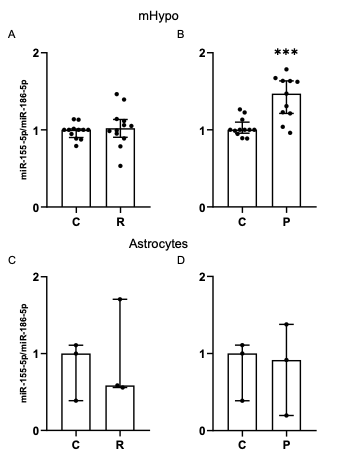


Figure S3: Resistin does not increase miR-155-5p expression in mHypo neuronal cell line nor astrocytes. mHypo neuronal cell line (A and B) and primary cultured mice astrocytes (C and D) were treated with placebo, resistin, or palmitate for 16 hours. Then, miR155-5p expression was measured by RT-qPCR and normalized to miR-186-5p. Data are median with interquartile range (n = 3 independent experiments, experiments were performed with 3 technical replicates for mHypo and 3 technical replicates for astrocyte primary culture). Significant difference at *** p<0,001.

Fig. S4


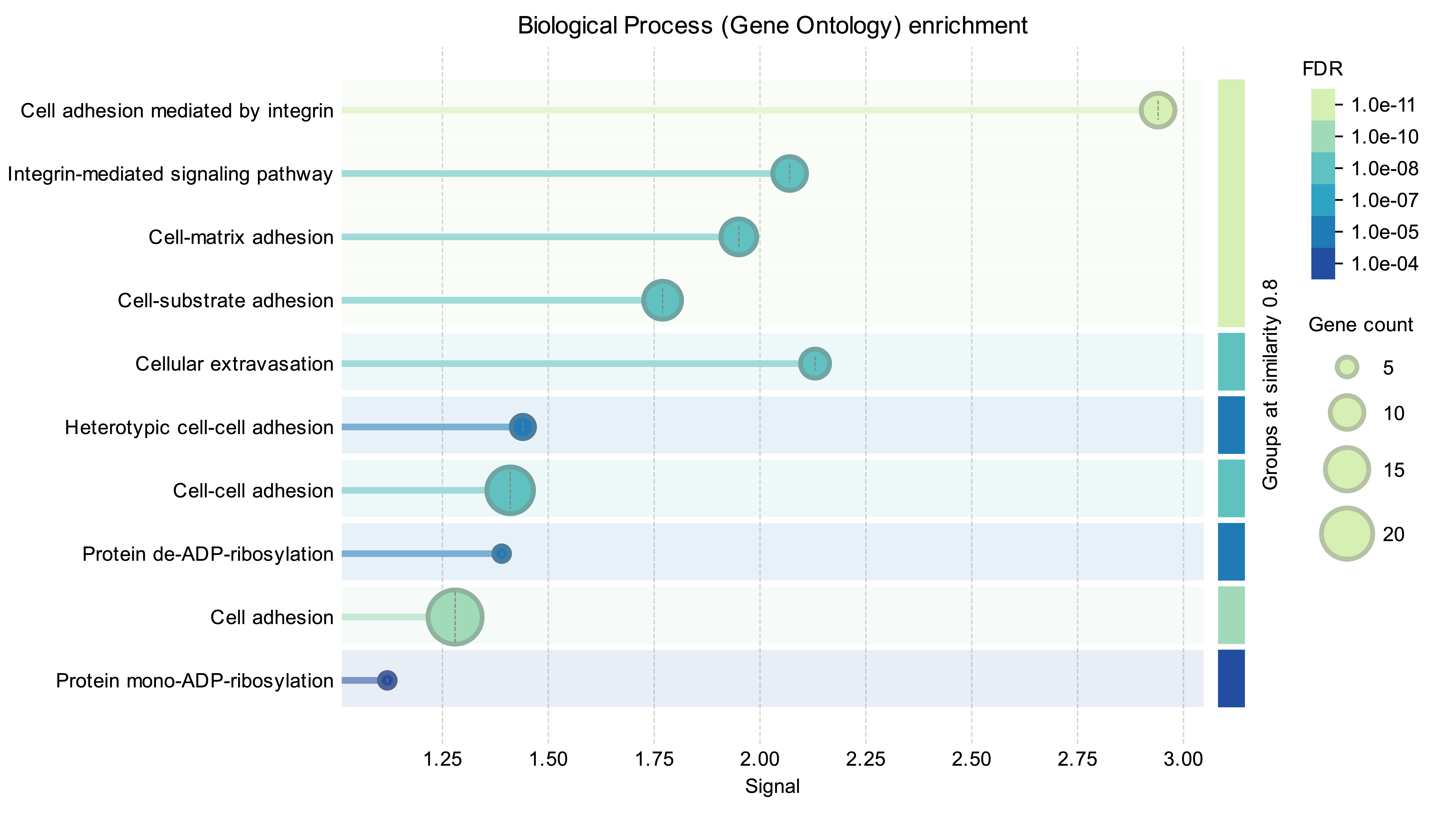

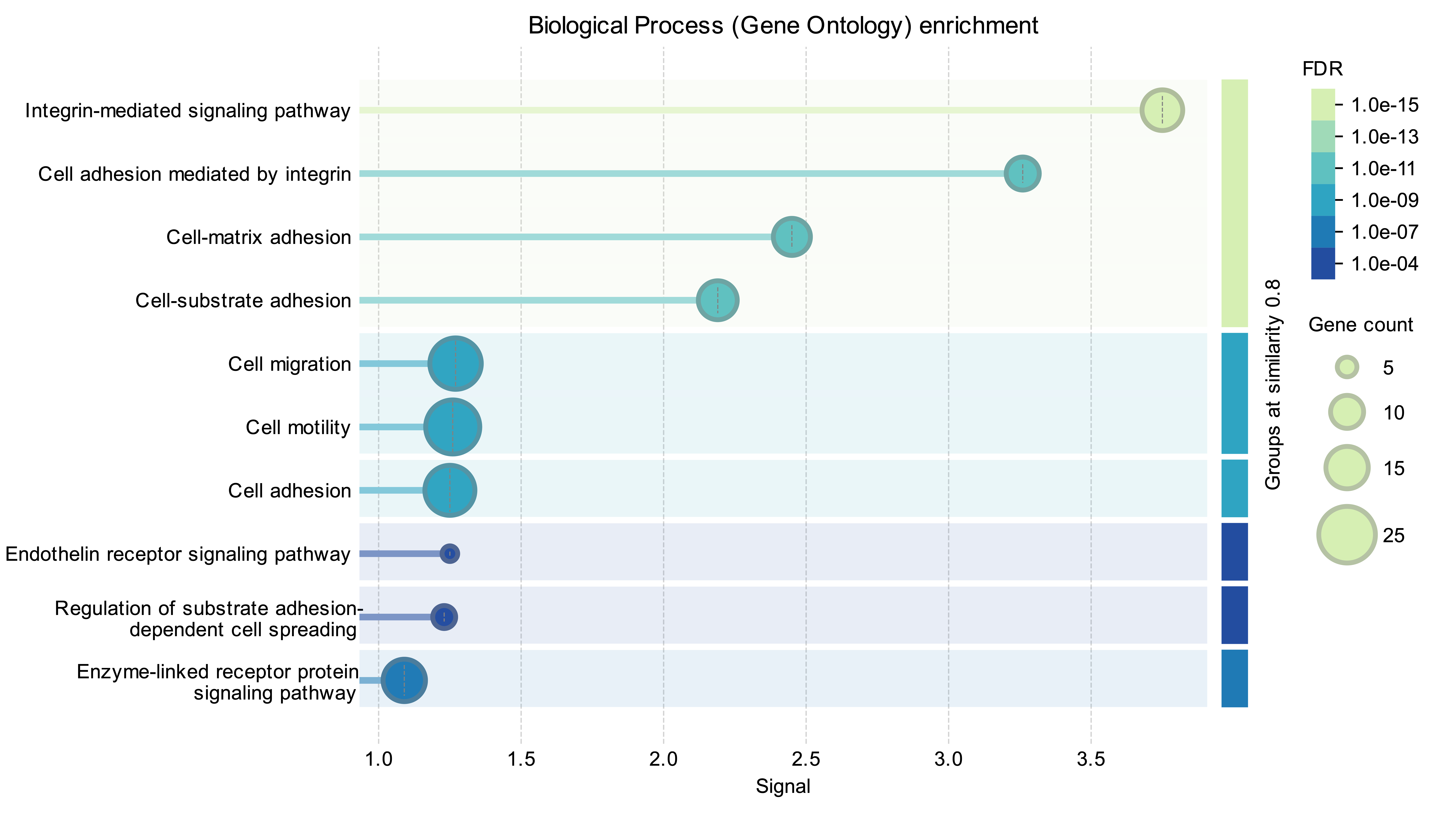


Males

Females

Figure S4. Schema showing biological process of the identified miR-155-5p targets (listed in table S3) using ShinyGO 0.82 (STRING 2.0) software.

Fig.S5

C

**Figure S5. Validation of miR-155-5p knockdown and co-localization with IBA1-positive microglia.**
(A) miRscope was used to detect and quantify miR-155-5p expression in the arcuate nucleus of *miR-155-5p^loxP/loxP*mice fed a high-fat diet (HFD) and treated with either control AAV8 or AAV8-Cre. miR-155-5p is shown in red, and nuclei are counterstained with DAPI (blue).
(B) Quantification of miR-155-5p puncta per surface unit. *p* < 0.05.
(C) Co-localization of miR-155-5p (green, detected by miRscope) with IBA1-positive microglia (red, detected by RNAscope using IBA1-specific probes). DAPI (blue) marks nuclei.

Table S1-1. Table extracted from the miRnome of mouse hypothalamus: Wild-type (WT, treated by ICV with placebo, WT Resistin (wild type treated by ICV with resistin), TLR-4KO (treated by ICV with placebo), and TLR-4KO Resistin (treated by ICV with resistin).

| miRNA Name | WT Placebo | WT Resistin | TLR-4KO Placebo | TLR-4 KO Resistin |
| --- | --- | --- | --- | --- |
| mmu-miR-7087-5p | 1 | 18.03 | 1.00 | 1.00 |
| mmu-miR-3963 | 1 | 6.45 | 1.00 | 1.00 |
| mmu-miR-21a-3p | 1 | 6.08 | 1.00 | 1.00 |
| mmu-miR-8101 | 1 | 5.92 | 1.00 | 1.00 |
| mmu-miR-155-5p | 1 | 5.81 | 1.00 | 1.00 |
| mmu-mir-6240-3p_novel | 1 | 4.67 | 1.00 | 10.87 |
| mmu-miR-1291 | 1 | 4.63 | 1.00 | 1.00 |
| mmu-miR-2137 | 1 | 3.77 | 1.00 | 1.00 |
| mmu-miR-5099 | 1 | 3.27 | 1.00 | 1.00 |
| mmu-miR-146a-5p | 1 | 3.02 | 1.00 | 2.22 |
| mmu-miR-6937-5p | 1 | 0.14 | 1.00 | 1.00 |
| mmu-miR-29b-1-5p | 1 | 0.03 | 1.00 | 1.00 |
| mmu-miR-96-5p | 1 | 1.00 | 2065.11 | 1.00 |
| mmu-miR-183-5p | 1 | 1.00 | 1101.91 | 1.00 |
| mmu-miR-211-5p | 1 | 1.00 | 529.92 | 1.00 |
| mmu-miR-184-3p | 1 | 1.00 | 247.15 | 1.00 |
| mmu-miR-182-5p | 1 | 1.00 | 146.73 | 1.00 |
| mmu-miR-122-5p | 1 | 1.00 | 54.47 | 1.00 |
| mmu-miR-6240 | 1 | 1.00 | 1.00 | 329.05 |
| mmu-miR-6238 | 1 | 1.00 | 1.00 | 211.64 |
| mmu-miR-3963 | 1 | 1.00 | 1.00 | 6.82 |
| mmu-miR-141-3p | 1 | 1.00 | 1.00 | 5.38 |
| mmu-miR-99a-5p | 1 | 1.00 | 1.00 | 2.85 |
| mmu-miR-124-3p | 1 | 1.00 | 1.00 | 2.76 |
| mmu-miR-30a-5p | 1 | 1.00 | 1.00 | 2.47 |
| mmu-miR-5106 | 1 | 1.00 | 1.00 | 0.11 |
| mmu-miR-7049-5p | 1 | 1.00 | 1.00 | 0.09 |

Table S2. Targets of miR-155-5p identified by HITS-CLIP in SIMA9 microglia cell line.

| Geneid |  |
| --- | --- |
| ENSMUSG00000000317 | Bcl6b |
| ENSMUSG00000022311 | Csmd3 |
| ENSMUSG00000024201 | Kdm4b |
| ENSMUSG00000026971 | Itgb6 |
| ENSMUSG00000027965 | Olfm3 |
| ENSMUSG00000040725 | Hnrnpul1 |
| ENSMUSG00000045095 | Magi1 |
| ENSMUSG00000046500 | Tafa4 |
| ENSMUSG00000049252 | Lrp1b |
| ENSMUSG00000085779 | Atcayos |
| ENSMUSG00000033420 | Antxr1 |
| ENSMUSG00000029338 | Antxr2 |
| ENSMUSG00000041112 | Elmo1 |
| ENSMUSG00000048249 | Crebrf |
| ENSMUSG00000027189 | Trim44 |
| ENSMUSG00000059173 | Pde1a |
| ENSMUSG00000062078 | Qk |
| ENSMUSG00000063052 | Lrrc39 |

Table S3. Targets of miR-155-5p identified by HITS-CLIP in the hypothalamus of male and female mice.


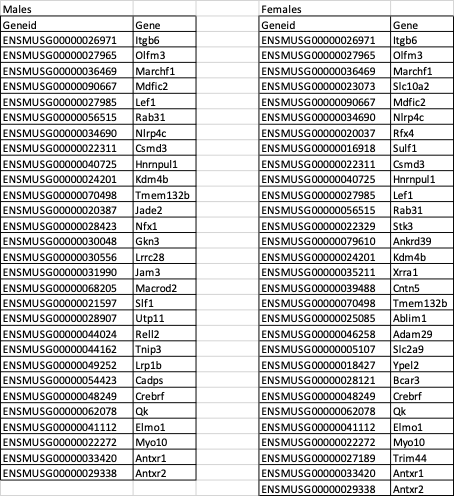


Table S4. List and sequence of the used primers.
